# GPR30 as an initiator of tamoxifen resistance in hormone-dependent breast cancer

**DOI:** 10.1186/bcr3581

**Published:** 2013-11-29

**Authors:** Zhiqiang Mo, Manran Liu, Fangfang Yang, Haojun Luo, Zhenhua Li, Gang Tu, Guanglun Yang

**Affiliations:** 1 Department of Endocrine Surgery, The First Affiliated Hospital of Chongqing Medical University, Youyi road 1, Chongqing, Chongqing 40016, China; 2 Key Laboratory of Laboratory Medical Diagnostics, Chinese Ministry of Education, Chongqing Medical University, Chongqing, China; 3 Department of Internal Medicine, Shenzhen Children’s Hospital, Shenzhen, China

## Abstract

**Introduction:**

Tamoxifen is widely used to treat hormone-dependent breast cancer, but its therapeutic benefit is limited by the development of drug resistance. Here, we investigated the role of estrogen G-protein coupled receptor 30 (GPR30) on Tamoxifen resistance in breast cancer.

**Methods:**

Primary tumors (PTs) of breast cancer and corresponding metastases (MTs) were used to evaluate the expression of GPR30 and epidermal growth factor receptor (EGFR) immunohistochemically. Tamoxifen-resistant (TAM-R) subclones derived from parent MCF-7 cells were used to investigate the role of GPR30 in the development of tamoxifen resistance, using MTT assay, western blot, RT-PCR, immunofluorescence, ELISA and flow cytometry. TAM-R xenografts were established to assess anti-tumor effects of combination therapy with GPR30 antagonist G15 plus 4-hydroxytamoxifen (Tam), using tumor volume measurement and Terminal deoxynucleotidyl transferase dUTP nick end labeling (TUNEL).

**Results:**

In 53 human breast cancer specimens, GPR30 expression in MTs increased compared to matched PTs; in MTs, the expression patterns of GPR30 and EGFR were closely related. Compared to parent MCF-7 cells, TAM-R cells had greater growth responses to 17β-estradiol (E2), GPR30 agonist G1 and Tam, and significantly higher activation of Mitogen-activated protein (MAP) kinases; but this increased activity was abolished by G15 or AG1478. In TAM-R cells, GPR30 cell-surface translocation facilitated crosstalk with EGFR, and reduced cAMP generation, attenuating inhibition of EGFR signaling. Combination therapy both promoted apoptosis in TAM-R cells and decreased drug-resistant tumor progression.

**Conclusions:**

Long-term endocrine treatment facilitates the translocation of GPR30 to cell surfaces, which interferes with the EGFR signaling pathway; GPR30 also attenuates the inhibition of MAP kinases. These factors contribute to tamoxifen resistance development in breast cancer. Combination therapy with GPR30 inhibitors and tamoxifen may provide a new therapeutic option for drug-resistant breast cancer.

## Introduction

Tamoxifen is commonly used as an anti-estrogen treatment for patients with hormone-dependent breast cancer [1,2]. Although most patients benefit from this therapy, approximately 50% of responsive tumors eventually relapse due to development of tamoxifen resistance [3,4]. Acquired tamoxifen resistance is a crucial therapeutic problem for which several molecular mechanisms have been proposed to be responsible [5].

Tamoxifen resistance mechanisms are complex. Inappropriate activation of the epidermal growth factor receptor (EGFR) signaling pathway readily promotes anti-hormonal treatment failure in breast cancer [6-8]; EGFR over-expression reportedly decreases sensitivity to endocrine therapy in breast cancer patients [9]. EGFR downstream elements, which directly stimulate proliferative and survival signaling, are extraordinarily active in tamoxifen-resistant (TAM-R) cells [10-12]. These pivotal intermediates can also phosphorylate the AF-1 domain on estrogen receptor (ER) protein, transforming the tamoxifen–ER complex into a positive nuclear transcription factor [13]. However, initial mechanisms of increased EGFR activation are still undefined.

The G-protein coupled receptor 30 (GPR30), a seven-transmembrane domain protein, was recently identified as a novel estrogen receptor structurally distinguished from the classic ERα and ERβ [14]. The selective ER modulator tamoxifen, its metabolites, 4-hydroxytamoxifen (Tam), estrogen or the pure anti-estrogen fulvestrant, acting as a GPR30 agonist, could induce rapid non-genomic effects in breast cancer cells [15]. Reportedly approximately 50% of breast cancer patients express GPR30, which is consistent with development of tamoxifen resistance [16,17]. In breast cancer cells, estrogen activated-GPR30 cleaves into Gα and Gβγ. The Gβγ subunit, which modulates nongenomic signaling events, increases SRC-like tyrosine kinase activation, leading to phosphorylation of adaptor protein SHC by activating metalloproteases; this results in extracellular release of heparin-bound epidermal growth factor (HB-EGF) [18-20]. Release of HB-EGF can stimulate the EGFR signaling pathway, leading to induction of Erk1/2 phosphorylation [20]. Interestingly, the Gα subunit attenuates Erk1/2 activity via inhibitory activation of protein kinase A on RAF1 through cAMP generation [18,21]. Inhibition and stimulation of Erk1/2 are mediated by estrogen in breast cancer cells [18,20,21]. Here, we hypothesized that tamoxifen activates crosstalk between the GPR30 and the EGFR signaling pathway, while suppressing ER activation in GPR30/ER + breast cancer patients. As GPR30/EGFR crosstalk intensifies under endocrine therapy, breast cancer develops tamoxifen resistance due to growth stimulation induced by EGFR signaling.

We found that in 73.58% (39/53) of metastasis (MT) specimens, GPR30 expression, which is associated with EGFR expression, increased compared to their corresponding primary tumors (PTs). In MCF-7 cells, Tam treatment causes GPR30 to translocate to the cell surface, where it interacts with the EGFR signaling pathway. Moreover, GPR30 also reduces cAMP generation which, in turn, attenuates cAMP’s inhibition of EGFR downstream elements. Combination therapy with GPR30 inhibitor and Tam could promote initiation of apoptosis in TAM-R cells, while discouraging drug-resistant xenograft progression. Together, our results suggest that GPR30 interference with the EGFR signaling pathway is an initial factor in development of tamoxifen resistance in breast cancer.

## Methods

### Materials

All chemicals and antibiotics for cell culture were purchased from Beyotime (Haimen, China). Tam, 17β-estradiol (E2), dimethyl sulfoxide (DMSO) and 3-(4, 5-dimethylthiazol-2-yl)-2, 5-diphenyltetrazolium bromide (MTT) were obtained from Sigma-Aldrich (Steinheim, Germany). GPR30 agonists G1 and antagonist G15 were purchased from Tocris (Ellisville, USA). Rabbit anti-GPR30 polyclonal antibody was purchased from Abcam (Cambridge, UK). Affinity-purified rabbit antibody against EGFR was obtained from Bio-world (Saint Louis Park, MN, USA). Fluorescein isothiocyanate 4′, 6-diamidino-2-phenylindole (DAPI), diaminobenzidine (DAB) detection and secondary antibody conjugated with horseradish peroxidase (HRP) were obtained from Zsbio (Beijing, China). (D)MEM, GPR30 antisense oligonucleotides and β-actin antisense oligonucleotides were purchase from Invitrogen (New York, US).

### Cell culture

Human MCF-7 breast carcinoma cells (MCF-7) were purchased from Institute of Biochemistry and Cell Biology, Chinese Academy of Sciences (IBCB, Shanghai, China) and routinely grown in (D)MEM containing 5% fetal bovine serum (FBS), 10 μg/ml insulin, 100 U/ml penicillin, and 100 μg/ml streptomycin. TAM-R sublines were isolated by exposing high-density MCF-7 cells to 1 × 10^-6^ M Tam for 30 days [22]. Matched control cells were obtained by culturing MCF-7 cells in medium containing 0.1% ethanol. To maintain drug resistance, TAM-R cells were grown continuously in (D)MEM supplemented with 5% FBS and 1 × 10^-7^ M Tam. All cell lines were cultured at 37°C in a humidified 5% CO_2_ atmosphere. Before all experiments, cells were switched to phenol red-free (D)MEM containing 0.5% charcoal-dextran–stripped FBS for two days, excepted where noted.

The experiments performed in this study do not required Institute Ethics Board approval, because only commercially available cell lines were used.

### Specimens

The 77 archival paraffin-embedded breast cancer specimens were obtained from the Clinical Diagnostic Pathology Center, Chongqing Medical University (Chongqing, China). All patients, who underwent surgery at the 1st Affiliated Hospital of Chongqing Medical University from 1999 to 2011 were diagnosed by the same center and were only treated with tamoxifen after surgery. Exclusion criteria included a previous history of adjuvant anti-hormonal or cytostatic treatment, primary non-operable tumor and incomplete follow-up data. Median age at the time of primary diagnosis was 50.6 years (range: 28 to 91 years). The follow-up was performed at the first recurrence of disease. The median follow-up time of the study population was 61 months (range: 1 to 144 months). All patients involved in this study consented to participate in the study and publication of its results. The experiments were approved by the Ethics Committee of the First Affiliated Hospital of Chongqing Medical University and were conducted in compliance with the Helsinki Declaration.

### Immunohistochemistry

Sections of paraffin-embedded breast cancer specimens were mounted on SuperFrost Plus Glass Slides (Zsbio, Beijing, China), heated overnight and prepared using a Streptavidin-Peroxidase Kit (Zsbio, Beijing, China) according to the manufacturer’s instructions. The slides were incubated with commercial rabbit anti-GPR30 polyclonal antibody diluted 1:250, and affinity-purified rabbit antibody against EGFR diluted 1:200, for 2 hours at 37°C, then exposed to horseradish peroxidase-conjugated goat anti-rabbit IgG for 20 minutes at 37°C. Reactions were visualized by DAB detection. Nuclei were counterstained with Mayer’s modified hematoxylin.

### Evaluation of GPR30 and EGFR staining results

A modified semi-quantitative scoring system was used to evaluate the intensity of immunoactive areas. Scores were applied as follows: staining extent was classified as: 0, negative staining in all cells; 1, <1% cells stained; 2, 1% to 10% of cells stained; 3, 11% to 40% cells stained; 4, 41% to 70% cells stained; 5, 71% to 100% cells stained. Staining intensity was classified as: 0, negative; 1, weak; 2, moderate; 3, strong. Extent and intensity scores were multiplied to give total immunohistochemical (IHC) scores, ranging from 0 to 8. GPR30+ expression was defined for specimens that scored ≥2.

For assessment of EGFR expression, scores were applied as follows: 0, no staining; 1, weak and incomplete staining of more than 10% of cells; 2, moderate and complete staining of more than 10% of cells; 3, strong and complete staining of more than 10% of cells.

### Growth assay

For these experiments, cells were seeded in 96-well plates at a density of 1 × 10^4^ cells per well. Two days later, the cells were treated with different concentrations of E2, G1 or Tam for five days with medium replacement on day three. The final concentration of vehicle (DMSO) was 0.1%. At the end of treatment, cells were incubated with 20 μl of 5 mg/ml MTT for four hours at 37°C under a culture hood. After removing medium, MTT solvent was added to each well for 15 minutes; a digital spectrophotometer was used to measure 590 nm optical density (OD) value, which was expressed as percent (%) of control.

### Immunofluorescent microscopy

For these experiments, cells were grown on sterile glass coverslips in 6-well plates at a density of 1 × 10^5^ cells per well. After 24 hours, cells were washed with cold PBS, fixed in paraformaldehyde for 20 minutes and permeabilized in 0.1% Triton for 15 minutes at room temperature. After background blocking with 5% goat serum in PBS for 30 minutes, cells were incubated with anti-GPR30 antibody overnight at 4°C. After incubation in primary antibody, secondary antibody conjugated with green fluorescent protein (GFP) was applied at room temperature for one hour. Excess antibody was removed by washing in PBS. Coverslips were mounted in vectashield with DAPI. For antibody specificity, cells incubated with secondary antibody served as controls. Cells were visualized using Nikon Phase Contrast Eclipse 80i. The images were collected using NIS-Elements software.

### RT-PCR

Total RNA was extracted from MCF-7 and TAM-R cells using RNAiso reagent (TaKaRa, Dalian, China) following the manufacturer’s instruction. cDNA was generated from total RNA via a PrimeScript RT reagent Kit (TaKaRa). To verify cDNA integrity and primer specificity, *GPR30* and *β-actin* were amplified by conventional PCR in an automatic Thermal Cycler using *GPR30* specific sense-primer, 5′-TCACGGGCCACATTGTCAACCTC-3′, antisense primer, 5′-GCTGAACCTCACATCTGACTGCTC-3′ and *β-actin* specific sense primer, 5′-TGACGTGGACATCCGCAAAG-3′, antisense primer, 5′-CTGGAAGGTGGACAGCGAGG-3′. The PCR amplified products were separated by electrophoresis in 1.5% agarose gels to visualize the products. Quantitative real-time PCR was conducted by Bio-Rad Miniopticom Real time PCR system using SYBR® Premix EX Taq™ II Kit (TaKaRa, Dalian, China). All the samples were amplified by real-time PCR twice and normalized to *β-actin*. Data were analyzed by comparison with a serial dilution series of cell cDNA.

### Immunoblotting

For these experiments, cells were cultured in 60-mm tissue culture plates at a density of 1 × 10^5^ cells per plate. Two days later, cells were treated as described in the figure legends for various times indicated in the results. Ethanol-treated cells were used as controls. After that, all the cells were washed with cold PBS and incubated on ice for five minutes with 200 μl lysis buffer (20 nM Tris (pH7.5), 150 nM NaCl, 1 mM β-glycerophosphate, 1 μg/ml leupeptin and aprotinin, 1 mM phenylmethanesulfonyl fluoride (PMSF)). Subcellular protein fractions were extracted using a Cell Membrane Protein Extraction Kit from Beyotime following the manufacturer’s instructions. All the samples were stored at -80°C until analysis.

Cellular proteins (50 μg) were boiled in SDS-PAGE sample loading buffer and separated on 10% SDS-PAGE. Proteins were electrotransferred onto polyvinylidene difluoride (PVDF) membranes using a Trans-Blot SD Semi-Dry Eletrophoretic Transfer Cell. The membranes were blocked overnight in Tris-buffered saline containing 0.1% Tween 20 and 10% defatted milk. Membranes were then incubated with primary antibodies as described in the figure legends for two hours at room temperature. Secondary antibody conjugated with HRP was used for a second incubation for one hour at room temperature. Bands of specific protein were visualized using chemiluminescent HRP substrate. Images were collected using a chemical luminescence imaging system.

### cAMP measurement

To measure intracellular cAMP, cells were seeded on 60-mm tissue culture plates at a density of 1 × 10^6^ cells per well. After 24 hours, cells were switched to a serum-starved, phenol red-free (D)MEM medium for 5 hours and then treated with E2, G1 or Tam as described in the figure legends. After treatment, cells were washed with PBS twice and frozen and thawed three times. The final concentrations of cAMP were quantified using an Enzyme Immunoassay Kit (R&D System, Minneapolis, MN, USA) according to the manufacturer’s instructions. Data were analyzed by measuring OD 590 values.

### Cell apoptosis analyses

For these experiments, cells were seeded on 6-well plates at a density of 1 × 10^5^ cells per well. Two days later, cells were treated with ethanol, Tam, G15, or G15 plus Tam for 48 hours. At the end of the treatment, cells were washed with PBS twice and collected by centrifuging at 2,000 rpm for five minutes. Cells were prepared by sequential addition of 500 μl binding buffer, 5 ml annexin V-FITC and 5 μl propidium iodide following the manufacturer’s instructions (Keygenbio, Nanjing, China). Data were analyzed using a BD FACSCalibur.

### Breast cancer xenograft models

TAM-R xenograft models were established in female ovariectomized athymic four- to six-week old nude mice (Animal Experimental Center of Chongqing Medical University, Chongqing, China) by implanting 5 × 10^6^ cells into mammary fat pads. Experiments were conducted in accordance with guidelines on animal care and use established by the Chongqing Medical University Experimental Animal Management Committee. The Ethics Committee of Chongqing Medical University approval was obtained for the study. When tumors grew to 150 to 200 mm^3^ (five to six weeks), the animals were randomly assigned to experimental groups at n = 5 per group. Tam and G15 were dissolved in absolute ethanol and diluted to the proper concentration with ethanol. For treatment with these compounds, 10 μL was added to 90 μL aqueous vehicle (0.9% NaCl with 0.1% albumin and 0.1% Tween-20). The control group received 10 μL ethanol alone added to 90 μL aqueous vehicle. Mice were given a subcutaneous injection (0.1 ml/mouse) of ethanol, Tam (50 μg), G15 (4 μg) or G15 (4 μg) plus Tam (50 μg) once daily. Tumor volumes were measured with a vernier caliper and calculated as 1/2 × length × width^2^ for tumors derived from TAM-R cell implants (Additional file 1: Table S1). At the end of the 56-day treatment, tumors were removed and embedded in paraffin. To assay the inhibitory effects of the treatment, sections were studied using an In Situ Cell Death Detection Kit (Zsbio, Beijing, China) following the manufacturer’s instruction. Samples were analyzed under a fluorescence microscope.

### Statistical analysis

The results are expressed as the means of three determinations ± SD. Curve fittings were performed with the Prism program (Graph Pad Software, San Diego, CA, USA). Statistical analysis was carried out using Student’s *t* test for paired observations. When three or more means were compared, analysis of variance was applied using the Prism program. Results were considered statistically significant if *P*<0.05.

## Results

### Expression of GPR30 and EGFR in breast cancer tissues

**Figure 1:**
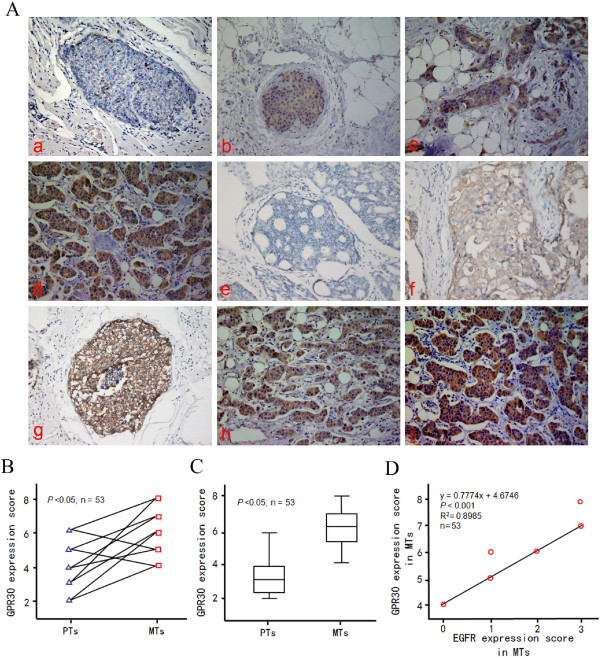
**Paraffin-embedded breast cancer tissue immunostained with GPR30 or EGFR antibodies.** The predominant staining pattern of GPR30 was cytoplasmic in carcinoma tissue, whereas EGFR was mainly expressed on plasma membrane **(A)**. Cytoplasmic GPR30 immunostaining of breast tumors was (a) negative, (b) weak, (c) moderate or (d) strong. EGFR staining of breast tumors was (e) no staining, (f) moderate and complete staining, or (g) strong and complete staining. GPR30 moderately stained primary tumors (h), but strongly stained corresponding tissue during tamoxifen treatment (i). Quantitative **(B)** and paired **(C)** expression of GPR30 was compared in 53 matched tissues from primary tumors (PTs) and their corresponding metastases (MTs). Pair-wise scatter plots showed the correlation of GPR30 and EGFR expression in MTs **(D)**. EGFR, epidermal growth factor receptor; GPR30, G-protein coupled receptor 30.

According to the inclusion criteria, breast cancer tissue specimens from 77 patients were eligible for analysis. Patients were considered GPR30^+^ if they had an IHC score of at least 2. GPR30 was predominantly expressed on plasma membranes and in cytoplasm, whereas EGFR was localized to plasma membranes in tumor tissues (Figure 1). GPR30 immunostaining patterns in breast cancer tissue were negative (Figure 1a), slightly positive (Figure 1b), moderately positive (Figure 1c), and strongly positive (Figure 1d).

Sites of recurrence included 29 local and 48 distant metastatic lesions; of these, 68.83% (53/77) of the paraffin-embedded breast cancer specimens were classified as GPR30^+^. To determine the relationship between GPR30 and tamoxifen resistance, GPR30 expression was detected in PTs and their corresponding MTs. In 53 tumors that recurred during treatment with tamoxifen, GPR30 expression was increased in 73.58% (39/53), decreased in 5.66% (3/53) and unchanged in 20.76% (11/53) (Figure 1B). As shown in Figure 1C, the mean IHC score for GPR30 was 3.46 ± 1.07 in PTs and 6.23 ± 0.91 in MTs, respectively (*P*<0.05). Also, in 77 MTs assessed for EGFR, 61.03% (47/77) were EGFR + and 74.46% (35/47) showed EGFR overexpression; and in 53 MTs (GPR30+), GPR30 expression was positively correlated with EGFR expression (R^2^ =0.8985, *P*<0.001).

### Therapeutic concentration of tamoxifen alters MCF-7 cell sensitivity to E2, G1 and Tam

Tam was tested on MCF-7 cells to assess variation in their proliferative potential during endocrine therapy. Acute exposure of MCF-7 cells to a therapeutic concentration of Tam (1 × 10^-6^ M) caused massive cell death over 5 days in medium supplemented with 5% FBS; however, the cytocidal effect of Tam was significantly diminished in those cells that survived after 21 days of continuous exposure to Tam. Exposure to 0.1% ethanol over a 21-day period did not change the inhibitory action of Tam (data not shown). Cells treated with Tam for 21 days, showed strong resistance to the therapeutic concentration of Tam and were termed TAM-R cells.

**Figure 2:**
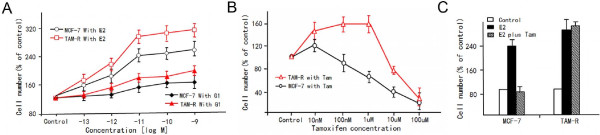
**Effects of 17ß-estradiol (E2), GPR30 agonist G1, and Tam on proliferation of parental MCF-7 cells and tamoxifen-resistant (TAM-R) cells. (A)** Cells were counted five days after treatment with the indicated concentrations of E2 and G1. **(B)** Cells were counted five days after treatment with different concentrations of Tam. **(C)** Cells were treated with 1 μM Tam in the presence of 10 nM E2 and counted after five days. Each experiment was repeated at least three times; results are expressed as means ± SD from three independent experiments. GPR30, G-protein coupled receptor; Tam, 4-hydroxytamoxifen.

Growth effects of E2, G1 and Tam were investigated in phenol-red free medium containing sufficient growth factors to support growth of cells. As expected, a low concentration of E2 effectively promoted MCF-7 cell growth; however, TAM-R cells showed more sensitivity to E2 growth stimulating effects. In contrast, a high concentration of the GPR30-specific agonist G1 stimulated only slight growth in MCF-7 cells, but gave significantly enhanced proliferative effects on TAM-R cells (Figure 2A). Although a low Tam concentration inhibited MCF-7 cell growth, TAM-R cell growth could be stimulated despite the presence of Tam (Figure 2B), showing that endocrine treatment significantly altered the pattern of response to Tam. Consistent with this observation above, the growth response of TAM-R cells to E2 (1 × 10^-10^ M) was 30% higher than MCF-7 cells, and this growth stimulation by E2 could be suppressed completely by 1 × 10^-6^ M Tam in MCF-7 cells, whereas it did not significantly inhibit the proliferation of TAM-R cells.

Tam treatment not only shifted E2 and G1 dose–response curves to the left, but also significantly altered patterns of response to Tam, thus contributing to the development of tamoxifen resistance in MCF-7 cells (Figure 2).

### Growth stimulations of TAM-R cells in response to E2, G1 and Tam were related to increased activation of MAP kinases

Activation of EGFR downstream elements, such as mitogen-activated protein kinases (MAPK) and phosphatidylinositol 3-kinase (PI-3 K), is an important mechanism of tamoxifen resistance. Also, the extra-cellularly regulated protein kinases-1 and -2 (Erk1/2) are part of a major MAPK pathway cascade, which mediates mitogenesis in hormone-sensitive breast cancer cells. To study associations between EGFR activation and increased responses to E2, G1 and Tam after tamoxifen resistance development, Erk1/2 phosphorylation levels were assayed.

**Figure 3:**
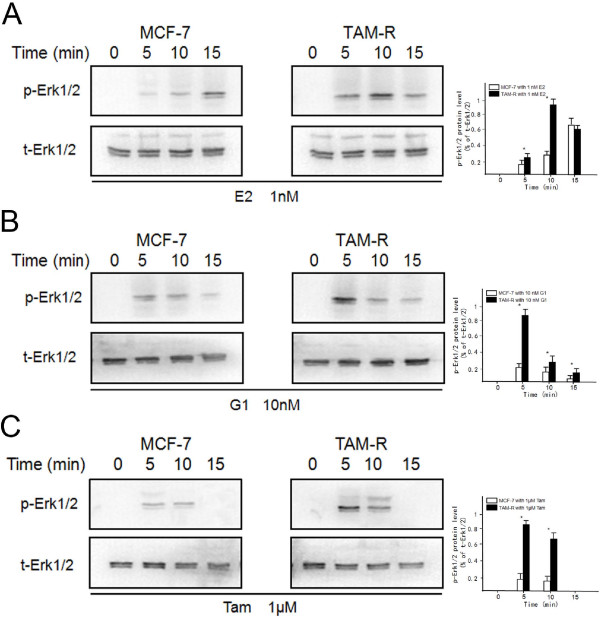
**Activation of Erk1/2 responses to E2, G1 or Tam in MCF-7 and TAM-R cells.** Erk1/2 expression was investigated by western blot using specific antibodies against phosphorylated (p) and total (t) Erk1/2 protein. Cells were cultured for the indicated times with 1 nM E2 **(A)**, 10 nM G1 **(B)** or 1 μM Tam **(C)** before preparation of cell lysates and western blot analysis. t-Erk1/2 expression was used as loading control. E2, 17β-estradiol; Tam, 4-hydroxytamoxifen; TAM-R, tamoxifen resistant.

E2 treatment can induce Erk1/2 phosphorylation, but patterns of phosphorylated-Erk1/2 (p-Erk1/2) differed distinctly between MCF-7 and TAM-R cells. In TAM-R cells, E2 induced p-Erk1/2 at 5 to 15 minutes, peaking at 10 minutes; in MCF-7 cells, Erk1/2 phosphorylation was more gradual, at 5 to 15 minutes after E2-incubation (Figure 3A).

TAM-R cells displayed higher Erk1/2 activation compared to MCF-7 cells during G1 treatment (Figure 3B). In TAM-R cells, earlier and significantly increased levels of p-Erk1/2 were seen at 5 minutes, and decreased at 10 to 15 minutes. In contrast, G1-induced Erk1/2 phosphorylation in MCF-7 cells was much weaker at 5 to 10 minutes than in TAM-R cells.

Similarly, Tam treatment also mediated rapid phosphorylation of Erk1/2 in MCF-7 and TAM-R cells (Figure 3C). In TAM-R cells, Tam can stimulate Erk1/2 activation, with peak increases at 5 and 10 minutes. Nevertheless, the activation of Erk1/2 induced by Tam was much weaker which started to decrease from 5 to 15 minutes in MCF-7 cells.

All these results indicate that increased agonistic effects of E2, G1 and Tam, which stimulated TAM-R cell proliferation, were related to inappropriate activation of Erk1/2, which was an EGF downstream factor.

### Increased Erk1/2activation was associated with intense GPR30/EGFR crosstalk in TAM-R cells

Because activated GPR30 at the cell membrane promotes HB-EGF release to activate the EGFR signaling pathway, resulting in phosphorylation of Erk1/2 in breast cancer cells, and TAM-R cells (as described above) increase activation of Erk1/2 in response to E2, G1 and Tam, the effect of GPR30 on EGFR signaling was tested in TAM-R cells.

**Figure 4:**
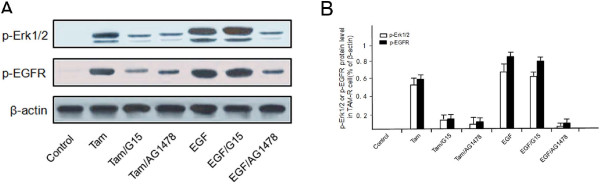
**Role of GPR30/EGFR signaling pathway in phosphorylation of Erk1/2 and EGFR in TAM-R cells.** Cells were treated with ethanol (control), 1 μM Tam or 10 ng/ml EGF alone or in combination with the GPR30 antagonist G15 or the EGFR inhibitor AG1478 for 10 minutes. Levels of p-Erk1/2 and p-EGFR were detected by western blot using specific antibodies **(A)**. Fold changes of expression were quantified, normalized to β-actin **(B)**. EGFR, epidermal growth factor receptor; GPR30, G-protein coupled receptor 30; Tam, 4-hydroxytamoxifen; TAM-R, tamoxifen resistant.

As shown in Figure 4, a strong phosphorylation of EGFR was observed in TAM-R cells, while Tam induced Erk1/2 phosphorylation. Coincidently, EGF could stimulate Erk1/2 and EGFR phosphorylation. In TAM-R cells, the GPR30-specific antagonist G15 could lower the levels of phosphorylated EGFR and Erk1/2 in the presence of Tam, but not in the presence of EGF. However, TAM-R cells pre-incubated with the EGFR inhibitor AG1478 could inhibit the ability of Tam or EGF to increase the activation of EGFR and Erk1/2.

These data suggest that inappropriate activation of Erk1/2 was related to the intense crosstalk of GPR30 to the EGFR signaling pathway during development of tamoxifen resistance.

### Translocation of GPR30 to cell surface facilitated GPR30/EGFR crosstalk in TAM-R cells

Because phosphorylation of Erk1/2 in TAM-R cells apparently depends on GPR30/EGFR crosstalk, we investigated the mechanism of the GPR30–EGFR interaction.

**Figure 5:**
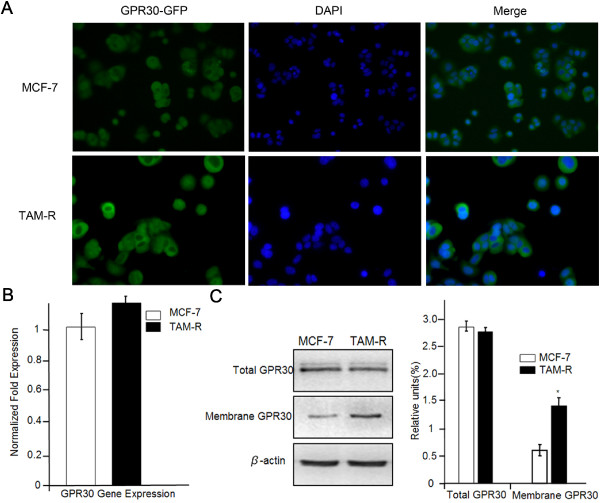
**Distribution and expression of GPR30 in MCF-7 and TAM-R cells.** The GPR30 subcellular location was detected using immunofluorescent staining. **(A)** Cells were stained with DAPI, GPR30-GFP alone or a combination (Merge). GPR30 mRNA and protein expressions in MCF-7 and TAM-R cells were quantified by qPCR **(B)** and western blot **(C)**. Fold changes of GPR30 in total protein and membrane-enriched protein fractions of MCF-7 and TAM-R cells were normalized to β-actin **(C)**. Each experiment was repeated at least three times. Results are expressed as means ± SD. *P <0.05 versus MCF-7 cells. DAPI, 4′, 6-diamidino-2-phenylindole; GPR30, G-protein coupled receptor 30; TAM-R, tamoxifen resistant.

As expected, green fluorescence was predominantly assembled in membrane and cytoplasm, indicating cellular locations of GPR30 in both MCF-7 and TAM-R cells. However, a variation was seen in TAM-R cells; whereas membrane and cytoplasm in MCF-7 cells were mildly stained, the degree of fluorescence was intensified in TAM-R cells (Figure 5A). It seemed that GPR30 expression significantly increased in TAM-R cells.

To quantify the level of GPR30, total GPR30 expression was studied in MCF-7 and TAM-R cells. *GPR30* mRNA levels relative to *β-actin* levels were quantified using RT-PCR and comparative Δt methods. There was no significant difference in mean *GPR30* mRNA levels between MCF-7 and TAM-R cells (Figure 5B) nor in relative expression of GPR30 protein normalized to β-actin in MCF-7 cells and TAM-R cells, as shown by western blot (Figure 5C). However, in enriched cytomembrane fractions of MCF-7 and TAM-R, a difference in GPR30 protein expression was clearly found. As shown in Figure 5C, the relative level of GPR30 in the membrane fraction of TAM-R was approximately 1.1 fold higher than in MCF-7 cells, indicating that a quantity of GPR30 had migrated to the cell membrane in TAM-R cells.

All these results reveal that GPR30, through cytomembrane translocation, enhances its interaction with EGFR, thus increasing Erk1/2 activation, leading to breast cancer proliferation during tamoxifen treatment.

### GPR30 attenuated inhibition of Erk1/2 activation by reducing cAMP in TAM-R cells

Although membrane translocation of GPR30 can enhance induction of EGFR downstream phosphorylation of Erk1/2 in TAM-R cells, counter-intuitively, the GPR30 subunit protein Gα can promote cAMP generation—which can attenuate Erk1/2 activation—by inhibiting activity of protein kinase A on RAF1. To elucidate the mechanism of GPR30 in stimulating Erk1/2 phosphorylation, intracellular cAMP production was measured by ELISA.

**Figure 6:**
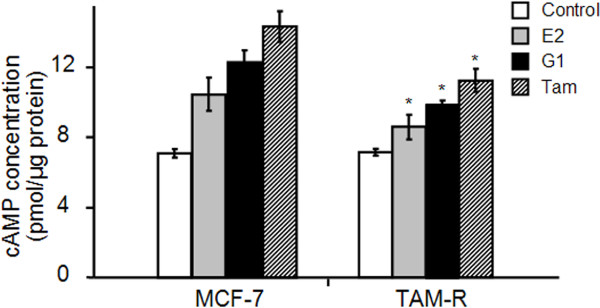
**Generation of cAMP mediated by GPR30 in MCF-7 and TAM-R cells.** Cells were incubated for five minutes with 1 nM E2, 10 nM G1 or 1 μM Tam; ELISA was then performed after preparation of cell lysates. Each experiment was repeated at least three times. Data show means ± SD. *P <0.05 versus similarly treated MCF-7 cells. E2, 17β-estradiol; GPR30, G-protein coupled receptor 30; Tam, 4-hydroxytamoxifen; TAM-R, tamoxifen resistant.

In MCF-7 cells, basal cAMP concentration [cAMP]_i_ was identical to that in TAM-R cells (Figure 6). In MCF-7 cells, E2 increased [cAMP]_i_ to 10.46 ± 0.94 pmol, G1 to 12.32 ± 0.65 pmol, and Tam to 14.33 ± 0.88 pmol (Figure 6A). In TAM-R cells, however, although rank orders of ligand-mediated cAMP production were the same as in MCF-7 cells, magnitudes of the increases were much less: E2 increased [cAMP]i in TAM-R cells to 8.59 ± 0.69 pmol, G1 to 9.96 ± 0.21 pmol, and Tam to 11.22 ± 0.66 pmol (Figure 6).

In TAM-R cells, GPR30 restricted its Gα subunit’s ability to promote cAMP generation, thus attenuating cAMP’s inhibition of Erk1/2 activation. GPR30 could, therefore, balance inhibition and stimulation of EGFR downstream elements that mediate Erk1/2 phosphorylation and promote tamoxifen resistance.

### GPR30/EGFR crosstalk mediated TAM-R cell survival

As enhanced interaction between GPR30 and EGFR signaling was seen to increase Erk1/2 phosphorylation in TAM-R cells, and Erk1/2 activates gene transcription leading to breast cancer proliferation, we investigated the role of GPR30/EGFR crosstalk in cell survival.

**Figure 7:**
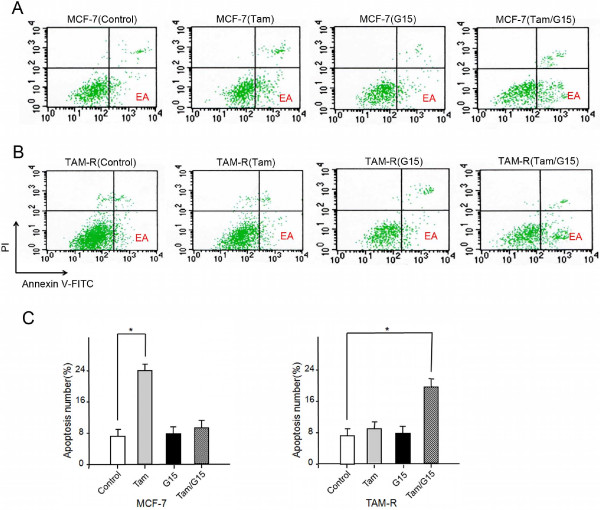
**Synergistic effects of GPR30 antagonist G15 and Tam on apoptosis.** Cells were treated with ethanol (control), 1 μM Tam, 10 nM G15 alone or 10 nM G15 plus 1 μM Tam for 48 hours using Annexin V-FITC flow cytometry. Scattergrams show numbers of MCF-7 **(A)** and TAM-R **(B)** cells in early-phase apoptosis (EA) using the indicated treatments. **(C)** Histograms show percentages of MCF-7 (A) and TAM-R (B) cells in early-phase apoptosis 48 hours after using the indicated treatments. Data show means ± SD from three independent experiments. * *P*<0.05, versus control; ** *P*<0.05 versus Tam treatment. GPR30, G-protein coupled receptor 30; Tam, 4-hydroxytamoxifen; TAM-R, tamoxifen resistant.

Among MCF-7 cells, Tam-treated cells stayed in early-phase apoptosis relative to ethanol-treated cells (Figure 7A), which is consistent with a study showing that tamoxifen and its active metabolites inhibit cell survival by inducing early-phase apoptosis [2]. In contrast, the Tam-treated, G15-treated or G15/Tam-treated groups did not significantly differ in the percentage of cells in early-phase apoptosis (Figure 7A). However, G15/Tam treatment induced some TAM-R cells to stay in early-phase apoptosis, unlike Tam or G15 alone (Figure 7B). The percentage of cells in early-phase apoptosis in each group was quantified (Figure 7C). In MCF-7 cells, Tam treatment led to 14.31 ± 0.35% increase in early-phase apoptosis compared to ethanol-treated cells. Although Tam or G15 alone did not significantly induce apoptosis in TAM-R cells, when combined, they induced 10.63 ± 1.21% increase in early-phase apoptosis.

These results indicate that GPR30 crosstalk with EGFR signaling is crucial to the anti-cytocidal effect of tamoxifen, which impels MCF-7 cells to develop tamoxifen resistance.

### GPR30 inhibitor G15 improved TAM-R xenograft response to endocrine treatment

Because GPR30 influences TAM-R cell survival by interacting with EGFR signaling under Tam exposure, effects of combined therapy with the GPR30 specific antagonist G15 and Tam on tamoxifen-resistant xenografts was studied.

**Figure 8:**
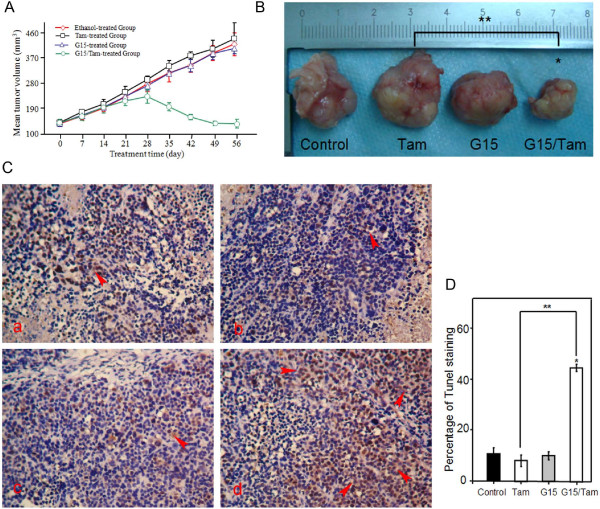
**Assessing the therapeutic effect of combination therapy with Tam plus G15 in a TAM-R xenograft.** Nude mice bearing TAM-R tumors were randomized on day 0 to receive ethanol alone, Tam (50 μg) alone, G15 (4 μg) alone or G15 (4 μg) in combination with Tam (50 μg) **(A)**. Images represent the xenograft tumors in monotherapy and combination therapy groups **(B)**. Paraffin-embedded sections from ethanol alone (a), Tam alone (b), G15 alone (c) or G15 in combination with Tam (d) were labeled for cellular apoptosis using Tunel **(C)**. Histogram shows percentage of apoptotic tumor cells induced by monotherapy or combination therapy for xenograft experiments **(D)**. *P<0.05 versus ethanol alone; ** *P* <0.05 versus Tam alone. Results are expressed as means ± SD. Tam, 4-hydroxytamoxifen; TAM-R, tamoxifen resistant.

Tamoxifen-resistant tumors were visible by 35 to 42 days in female ovariectomized athymic nude mice. In these experiments, the mean volume of ethanol-treated tumors (control group) increased by 3.2-fold over 56 days, whereas the mean volumes of Tam-treated or G15-treated tumors did not significantly differ from the control group. However, combined treatment remarkably inhibited growth in tamoxifen-resistant xenografts during the intervention (Figure 8A). At the end of treatment, the combination group had approximately two-fold reductions in tumor volume compared to controls (Figure 8B). Moreover, this inhibition showed no obvious toxicity, as body weight did not greatly change.

To investigate the anti-tumor effect of the target treatment, growth inhibition was analyzed using paraffin sections of TAM-R xenograft by TUNEL assay. In TAM-R xenografts ethanol-treated (a), Tam-treated (b) and G15-treated (c) cells showed slight staining by TUNEL, but combination treatment (d) caused strong staining (Figure 8C); percentages of TUNEL staining were quantified (Figure 8D). In control cells, ethanol treatment caused 11.03 ± 1.01% apoptosis in TAM-R tumors; this result is supported by those of Massarweh *et al.*, which indicated that low estrogen levels result in a partial regression of hormone-dependent breast cancer due to induction of apoptosis [23]. The Tam- or G15-treated groups also induced apoptosis in tumors of 8.17 ± 0.67% or 13.27 ± 1.31%, respectively. These observations correspond with previous tumor volume studies. As expected, combination therapy with GPR30 antagonist G15 plus Tam had a massive anti-tumor effect on TAM-R xenografts, by approximately three-fold over the control group.

These results imply that GPR30 is a stimulation factor in tamoxifen-resistant xenograft growth, and inhibiting GPR30 activation by targeted therapy could restore the curative effect of endocrine treatment to tamoxifen-resistant breast cancer.

## Discussion

In this study, we investigated the role of GPR30 in the development of tamoxifen resistance in hormone-dependent breast cancer. GPR30, a seven-transmembrane domain G-protein coupled receptor [24], is expressed in approximately 50% of breast cancer patients and is thought to induce rapid estrogen action in breast cancer cells [25,26]. Tamoxifen and its metabolites have been shown to stimulate breast cancer proliferation through GPR30 in these particular circumstances [27-29]. Taken together, these findings suggest that GPR30 promotes tamoxifen resistance in patients with breast cancer during endocrine treatment.

Preclinical and clinical studies have shown that patients with ER^+^ breast cancer that over-expresses EGFR and HER-2 have a lower sensitivity or shorter duration of response to hormone therapy [30]. Inappropriate activation of growth factor receptors, especially in the EGFR family, is reportedly responsible for development of tamoxifen resistance [5,31,32]. In breast cancer patients, EGFR-targeted therapy suppresses tamoxifen-resistant tumor progression [5]; however, the initial activator of the EGFR signaling pathway is disputed. Reportedly, approximately 50% of ER + breast cancer patients express GPR30, which coincides with the development of tamoxifen resistance [31,33]. In our study, expression of GPR30 was significantly increased in MTs relative to their corresponding PTs, and was also correlated with EGFR expression in MTs. We, therefore, hypothesized that further study of GPR30 would provide insight into the development of tamoxifen resistance.

GPR30 is thought to be a new membrane-bound estrogen receptor, which differs from the classical nuclear estrogen receptors α and β (ERα and ERβ) [34] and with a disputed role as a functional estrogen receptor in breast cancer cells. Many studies show that GPR30 collaborates with ER to transmit estrogen signaling; others suggest that GPR30 inhibits proliferation of ER^+^ breast cancer cells [27,35]. Our experiments found stimulation in wild-type MCF-7 cells by E2 to be stronger than G1. These results suggest that GPR30 plays a secondary role in estrogen-induced proliferation in parent cells. In TAM-R MCF-7 cells, the abilities of E2 and G1 to promote cell proliferation were significantly increased, and Tam approaching a clinically relevant concentration (1 μM) stimulated cell growth. Thus, we can conclude that the capacity of GPR30 to mediate estrogen action is significantly reinforced during development of tamoxifen resistance in breast cancer cells.

Some of the very first reports indicated that the Gβγ subunit protein of GPR30 greatly affects the GPR30/EGFR signaling pathway [36]. Downstream of GPR30 signaling, E2-induction leads to activation of the SRC-like tyrosine kinase and metalloproteinases which, in turn, stimulates extracellular release of HB-EGF, presumably through the Gβγ subunit protein [37,38]. Release of HB-EGF allows it to activate the EGFR signaling pathway, resulting in induction of Erk1/2 phosphorylation with consequent stimulation of cell growth [20]. As expected, E2, G1 or Tam stimulates phosphorylation of Erk1/2 in MCF-7 cells. Interestingly, a stronger and earlier phosphorylated Erk1/2 was observed in TAM-R cells during E2, G1 and Tam treatment, respectively, although there was no significant difference in basal levels of Erk1/2 between MCF-7 and TAM-R cells. Moreover, these increased activations of Erk1/2 were coincident with EGFR phosphorylation in TAM-R cells. The GPR30-specific antagonist G15 could significantly inhibit phosphorylation of Erk1/2 and EGFR as did the EGFR inhibitor AG1478. We noted that GPR30 activation increased ligand-dependent EGFR activity, leading to an Erk1/2-mediated transcriptional response, thus contributing to the development of tamoxifen resistance in breast cancer cells.

As these observations indicate, GPR30 interaction with the EGFR signaling pathway could be an important mechanism in the development of tamoxifen resistance in MCF-7 cells. In human breast cancer MTs, endocrine treatment increases expression of GPR30 compared to corresponding PTs. Further experiments showed that increased GPR30 expression mainly occurred in membranes of TAM-R cells, whereas the total GPR30 expression did not change. GPR30 seemed to enhance interaction with the EGFR signaling pathway through its translocation to the cell membrane.

Redistribution of ERα has been proposed as the mechanism of acquired tamoxifen resistance in breast cancer [39], but, this hypothesis is disputed. ERα protein has no hydrophobic transmembrane domains or membrane-localizing sequences [40], and any potential role of cytoplasmic ERα interaction in the EGFR pathway in developing tamoxifen resistance is unclear. ERα and EGFR expression in human breast cancer tissue are also inversely correlated [41]; ERα seems to repress EGFR in breast cancer cells [42].

On the other hand, the Gαs subunit of GPR30 has been suggested to be responsible for E2 stimulation of adenylate cyclase and the ensuing increase in cAMP generation in breast cancer cells [21]. Production of cAMP triggered by GPR30 can attenuate Erk1/2 activity by suppressing protein kinase A (PKA) on RAF1 [37]. It is likely that there is an exact balance between inhibition and stimulation of the Erk1/2 pathway in MCF-7 cells [43]. In our study, the basal cAMP level of MCF-7 cells was similar to that of TAM-R cells, but E2-induced, G1-induced or Tam-induced cAMP generation in TAM-R cells was significantly lower than in MCF-7 cells. These reductions of cAMP production which receded as a result of PKA inhibition led to increased activation of Erk1/2 in TAM-R cells. All these results, showing that GPR30 destroyed the exact balance mentioned above, would promote the development of tamoxifen resistance in MCF-7 cells during endocrine treatment, but the precise molecular mechanism to explain how GPR30 causes an imbalance between inhibition and stimulation of the Erk1/2 pathway induced by cAMP is unclear at the present time. Further studies are needed to investigate this process.

Several lines of evidence indicate that PP2, an inhibitor of non-receptor tyrosine kinase c-Src—a mediator of the EGFR signaling pathway—can abolish E2-induced Erk1/2 phosphorylation and, thus, inhibit MCF-7 cell growth [44]. In our study, GPR30 activation was inhibited by its specific antagonist G15, thus restraining proliferation of TAM-R cells by initiating apoptosis under Tam intervention. These results are supported by the investigation of Ignatov *et al*., which indicated that GPR30 anti-sense oligonucleotides could eliminate GPR30 ligand-mediated growth stimulation of TAM-R cells [45]. In the *in vivo* study of the proliferative potential of GPR30, combination therapy of G15 plus Tam significantly reduced TAM-R tumor size, whereas treatments with Tam or G15 alone did not. GPR30-target treatment could increase apoptosis in TAM-R xenografts, whereas apoptosis rates from Tam or G15 treatment do not significantly differ from that of the ethanol-treated group. Synergistic interaction of GPR30 and the EGFR signaling pathway enhances breast cancer proliferation, which allows tumor progression in the presence of tamoxifen.

While several endocrine-resistant breast cancer models are based on inappropriate activity of the EGFR signaling pathway [8,22,23], the present model shows variable activation of the EGFR downstream cascade [6]. Levels of phosphorylated Erk1/2 increased transiently in our TAM-R cells and in long-term tamoxifen-treated models reported by others [46,47]. In contrast, sustained Erk1/2 phosphorylation was observed in long-term estrogen-deprived MCF-7 cells [48]. These differences may relate to ways that breast cancer cells adapt to various endocrine treatments (drug therapy or estrogen deprivation) [41,49,50]. Although inappropriate activation of the EGFR signaling pathway is widely accepted as a key mechanism of tamoxifen resistance, the initial factor that transactivates EGFR is still disputed. Our study thus aimed to demonstrate the role of GPR30 in the development of tamoxifen resistance. In breast cancer MTs, GPR30 expression significantly increased relative to corresponding PTs and correlated with EGFR expression. Endocrine treatment caused increased ligand-dependent activation of the EGFR downstream element Erk1/2, with consequential growth stimulation—which would lead breast cancer cells to develop tamoxifen resistance. These phenomena were possibly related to translocation of GPR30 to the cytomembrane and reduction of GPR30-induced cAMP production. As crosstalk between GPR30 and the EGFR signaling pathway intensified, inhibited GPR30 activity could promote apoptosis initiation in drug-resistant cells in the presence of tamoxifen. Moreover, combination therapy with the GPR30 specific antagonist G15 plus tamoxifen both restrained tumor progression, and restored the cytocidal effect of tamoxifen in drug-resistant xenografts. Our results provide experimental evidence of the important role of GPR30 in the development of tamoxifen resistance, establishing a new therapeutic target to delay drug resistance or improve response to endocrine treatment in cases that develop tamoxifen resistance.

## Conclusions

**Figure 9:**
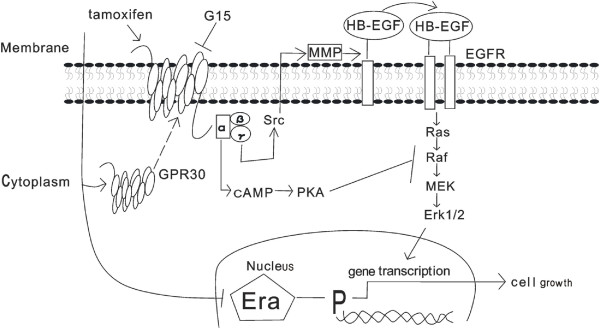
**Role of GPR30 in the development of tamoxifen resistance.** Long-term endocrine therapy can inhibit ERα-regulated gene transcription in hormone-dependent breast cancer; whereas tamoxifen-facilitated translocation of GPR30 to the cell membrane enhances crosstalk with EGFR signaling through the Gβγ subunit of GPR30. However, when treated with GPR30 plus tamoxifen, GPR30’s Gα subunit attenuates cAMP suppression of Erk1/2 phosphorylation of an EGF downstream factor. As tamoxifen is an agonist for GPR30, endocrine therapy can stimulate GPR30/EGFR crosstalk, leading to cell growth. When this activation effect exceeds ERα inhibition, breast cancer progresses under tamoxifen treatment. Interrupting direct crosstalk between GPR30 and EGFR by use of a GPR30-specific antagonist (G15) induces both cytocidal action *in vitro* and an antitumor effect *in vivo*. Targeted therapy with GPR30 could restore endocrine therapy response in tamoxifen-resistant breast cancer. EGFR, epidermal growth factor receptor; ER, estrogen receptor; GPR30, G-protein coupled receptor 30.

In summary, our findings suggest that long-term endocrine therapy facilitates translocation of GPR30 to cell membranes, resulting in inappropriate activation of the EGFR signaling pathway (Figure 9). Meanwhile, GPR30 attenuates the inhibitory effect of cAMP on MAP kinases. Combination treatment with the GPR30 specific antagonist G15 plus Tam induces both cytocidal action *in vitro* and antitumor progression *in vivo.* Thus, GPR30 might be a useful target in developing better treatments for TAM-R breast cancer patients.

## Abbreviations

(D)MEM: (Dulbecco’s) modified Eagle’s medium; DAB: Diaminobenzidine; DAPI: 4′, 6-diamidino-2-phenylindole; DMSO: Dimethyl sulfoxide; E2: 17β-Estradiol; EGFR: Epidermal growth factor receptor; ELISA: Enzyme-linked immunosorbent assay; Erk1/2: Extracellular-signal regulated kinase-1 and-2; ER: Estrogen receptor; GFP: Green fluorescent protein; GPR30: G-protein coupled receptor 30; HB-EGF: Heparin-bound epidermal growth factor; HRP: Horseradish peroxidase; IHC: Immunohistochemistry; MAPK: Mitogen-activated protein kinase; MT: Metastasis; MTT: 3-(4, 5-dimethylthiazol-2-yl)-2, 5-diphenyltetrazolium bromide; PBS: Phosphate-buffered saline; PT: Primary tumor; RT-PCR: Real time-polymerase chain reaction; Tam: 4-hydroxytamoxifen; TAM-R: Tamoxifen-resistant.

## Competing interests

The authors declare that they have no competing interests.

## Authors’ contributions

ZQM participated in the design of the study and carried out the immunoassays, molecular genetic studies and animal experiments and drafted the manuscript. MRL analyzed the data and helped to draft the manuscript. FFY performed the statistical analysis and participated in the sequence alignment. HJL carried out the immunoassays. ZHL participated in animal experiments. GLY conceived and designed the study. GT participated in study design and coordination. All authors read and approved the final manuscript.

## Supplementary Material

Additional file 1: Table S1 Detailed measurements of tumor volume of each individual TAM-R xenograft model. Table shows the length, width and volume of the mean and standard deviation (SD) of tumors in the Ethanol-treated (a), Tam-treated (b), G15-treated (c) and G15/Tam- treated (d) groups. The data were recorded weekly until the 56th week. Click here for file
