# Supplementary material for: GPR30 as an initiator of tamoxifen resistance in hormone-dependent breast cancer
Source: Breast Cancer Res. 2013 Nov 29;15(6):R114. doi: 10.1186/bcr3581 (PMC3978564; doi:10.1186/bcr3581)
Supplement: Additional file 1: Table S1 — Detailed measurements of tumor volume of each individual TAM-R xenograft model. Table shows the length, width and volume of the mean and standard deviation (SD) of tumors in the Ethanol-treated (a), Tam-treated (b), G15-treated (c) and G15/Tam- treated (d) groups. The data were recorded weekly until the 56th week. [file bcr3581-S1.pdf]

a

	Ethanol-treated Group					Average	SD
	**1**	**2**	**3**	**4**	**5**		
	**Length/Width**	**Length/Width**	**Length/Width**	**Length/Width**	**Length/Width**		
**0 day**	6.5/6.4	6.9/6.3	6.9/6.6	6.8/6.3	7.0/5.9	135.42	10.16
**7th day**	7.4/6.6	7.1/6.5	7.3/7.0	7.0/6.5	7.7/6.6	161.12	12.82
**14th day**	7.8/7.0	8.3/7.0	7.4/6.9	7.6/6.9	7.7/7.2	190.22	11.67
**21th day**	7.8/7.4	8.8/7.4	8.1/7.6	8.1/7.2	8.3/7.5	230.75	12.00
**28th day**	9.0/8.1	8.9/8.3	8.3/7.6	8.9/7.9	8.8/7.7	276.02	26.71
**35th day**	9.4/8.6	9.4/8.5	8.6/8.0	9.4/8.0	9.0/8.3	314.64	29.48
**42th day**	9.9/8.8	9.5/8.4	9.1/8.4	9.9/8.2	9.5/8.5	343.11	23.84
**49th day**	10.3/9.1	9.7/9.0	9.6/8.7	9.7/8.6	9.9/8.9	386.69	27.29
**56th day**	10.2/9.8	9.6/9.1	9.8/9.2	9.7/9.0	9.6/9.1	418.47	40.74

Measurement unit: millimeter

b

	Tam-treated Group					Average	SD
	**1**	**2**	**3**	**4**	**5**		
	**Length/Width**	**Length/Width**	**Length/Width**	**Length/Width**	**Length/Width**		
**0 day**	7.1/6.5	6.9/6.5	6.8/6.6	6.3/6.3	7.2/5.9	138.84	12.57
**7th day**	7.5/6.9	7.0/6.9	7.7/6.9	7.6/6.8	7.5/6.9	176.54	6.17
**14th day**	8.1/7.1	7.5/7.2	8.1/7.4	7.5/7.0	8.0/7.3	205.78	16.02
**21th day**	8.8/8.0	8.1/7.9	8.3/7.4	8.5/7.5	8.8/7.4	248.32	20.68
**28th day**	9.2/8.1	8.7/8.1	8.9/7.9	9.1/8.1	9.3/8.1	293.71	11.65
**35th day**	9.6/8.7	9.0/8.5	9.3/8.3	9.6/8.5	9.8/8.5	341.92	18.55
**42th day**	9.9/8.9	9.4/9.1	9.7/8.9	9.6/8.7	10.4/8.4	379.14	13.18
**49th day**	10.4/9.2	9.6/9.1	10.5/8.9	10.2/8.9	9.9/8.5	403.02	30.13
**56th day**	10.4/9.2	9.6/9.1	10.5/10.1	10.2/9.1	10.0/9.0	440.11	55.89

Measurement unit: millimeter

C

	G15-treated Group					Average	SD
	1	2	3	4	5		
	Length/Width	Length/Width	Length/Width	Length/Width	Length/Width		
**0 day**	6.7/6.6	7.2/6.4	6.4/6.3	6.8/6.2	6.6/6.2	135.59	10.27
**7th day**	6.9/7.0	6.8/6.6	7.2/6.9	7.7/6.7	7.0/6.8	164.64	10.17
**14th day**	7.4/7.4	7.3/7.2	7.7/7.3	7.7/6.9	7.5/7.0	192.81	10.42
**21th day**	7.9/7.8	8.0/7.5	8.3/7.5	8.7/7.6	7.7/7.3	231.04	17.37
**28th day**	8.3/7.9	8.7/8.0	8.5/7.9	8.9/8.1	8.4/7.8	270.03	15.05
**35th day**	8.9/8.3	9.1/8.4	9.0/8.3	9.0/8.3	9.0/8.5	314.55	8.05
**42th day**	9.1/8.5	9.0/8.5	9.5/8.7	9.3/8.4	9.6/8.8	342.64	21.46
**49th day**	9.8/8.9	10.0/9.0	10.3/8.8	9.9/8.8	9.3/8.7	385.45	20.58
**56th day**	10.0/9.1	10.2/9.0	10.5/9.0	9.9/8.9	9.3/9.0	404.23	19.53

Measurement unit: millimeter

d

	G15/Tam-treated Group					Average	SD
	**1**	**2**	**3**	**4**	**5**		
	**Length/Width**	**Length/Width**	**Length/Width**	**Length/Width**	**Length/Width**		
**0 day**	7.4/6.2	6.9/6.6	6.6/6.5	6.6/6.6	7.0/5.9	139.5	10.65
**7th day**	7.9/6.8	7.0/6.6	6.9/6.9	7.4/6.3	6.9/6.8	161.15	13.73
**14th day**	8.3/7.3	7.3/6/8	7.4/7.3	7.9/6.7	7.5/7.3	192.85	20.55
**21th day**	8.3/7.7	7.9/7.2	7.9/7.4	7.8/7.3	7.5/7.4	216.06	17.39
**28th day**	8.5/8.0	8.0/7.3	8.1/7.6	8.0/7.5	7.7/7.5	232.13	23.7
**35th day**	7.9/7.3	7.5/7.2	7.9/7.2	7.3/6.9	7.1/7.3	194.53	14.31
**42th day**	7.4/6.9	7.0/6.6	7.3/6.5	7.3/6.5	6.9/6.7	158.39	9.98
**49th day**	7.1/6.3	7.0/6.5	6.9/6.1	6.8/6.2	6.6/6.3	135.76	8.31
**56th day**	7.0/6.5	6.8/6.7	6.7/6.1	6.6/6.4	6.5/6.0	135.46	15.05

Measurement unit: millimeter
